# Psychosocial support for adult-onset type 1 diabetes: the living with and adapting to type 1 diabetes programme - a cross-national feasibility study

**DOI:** 10.3389/fcdhc.2026.1749766

**Published:** 2026-03-17

**Authors:** Mette Due-Christensen, Rhawann El Hakim, Daniel Piper, Ewa Romanczuk, Julie L. Wad, Kirsty Winkley, Ingrid Willaing, Bryan Cleal, Angus Forbes

**Affiliations:** 1 Florence Nightingale Faculty of Nursing, Midwifery and Palliative Care, King’s College London, London, United Kingdom; 2 Department of Prevention, Health Promotion and Community Care, Steno Diabetes Center Copenhagen, Herlev, Denmark; 3 Patient and Public Involvement Representative, London, United Kingdom; 4 Department of Endocrinology, Steno Diabetes Center Odense, Odense, Denmark

**Keywords:** adaptation, adult-onset type 1 diabetes mellitus, diagnosis, feasibility study, psychosocial

## Abstract

**Background:**

Psychosocial challenges related to adult-onset type 1 diabetes are not systematically addressed in routine diabetes care. The Living with and Adapting to DiabetEs pRogramme (LADDER) was developed to support psychosocial adaptation to diabetes through two distinct interventions: 1) one-to-one consultations, and 2) group sessions. The LADDER interventions were tested in Denmark and the UK to evaluate the feasibility of 1) the research processes; 2) the delivery of the intervention; 3) collection of psychosocial and clinical outcomes and perceived benefits of participation; and to 4) identify key areas for improvement of the interventions.

**Methods:**

We aimed to examine the feasibility of the one-to-one consultations via a controlled design with either non-random (Denmark) or random allocation (UK) to intervention or usual care and the group sessions via a wait-list randomised trial (UK). Psychosocial outcomes were collected through questionnaires, and clinical outcomes were obtained from electronic records. A concurrent process evaluation was conducted through interviews with participants and healthcare professionals.

**Results:**

The recruitment target was reached in Denmark. Due to a lower-than-expected number of participants, randomisation was abandoned in the UK. Questionnaire response rates were low, and clinical data were difficult to obtain. Due to uncontrolled study designs, small sample sizes, and high questionnaire attrition, quantitative comparisons between intervention and control groups were not feasible. Qualitative findings suggested that the LADDER interventions may support psychosocial adaptation by facilitating reflection, articulation of concerns, and shared understanding within supportive clinical and peer contexts.

**Conclusion:**

Conducting an evaluation of the LADDER interventions through a controlled study was not feasible, likely due to small study populations at each site, combined with changes in clinical working following COVID-19. We suggest using a realist evaluation approach for a future larger study to explore what aspects of LADDER works, for whom and in which contexts.

## Introduction

More than half of those diagnosed with type 1 diabetes (T1D) are adults (aged 18 years and older) (1, 2). A diagnosis of T1D in adulthood can be very disruptive, demanding the acquisition of challenging self-management skills and potentially generating fears related to the risk of hypoglycaemia and future diabetes complications (3, 4). These disruptions can be particularly challenging in adulthood, where people often have established their life in terms of employment, relationships, and lifestyle choices (5, 6). Therefore, people with adult-onset T1D experience unique psychological and social challenges following diagnosis (7), and these challenges can make the acceptance of the diagnosis and integration of diabetes self-management into everyday life and relationships difficult (8, 9). Impaired self-management have been shown to increase the risk of developing long-term complications (10, 11). In addition, some studies have reported an association between the presence of psychological challenges in the first five years after diagnosis and suboptimal HbA_1c_ levels (12, 13), suggesting that attending to these challenges early in their disease experience is important and may reduce diabetes complications and improve psychological well-being. There is a lack of evidence-based effective interventions addressing psychosocial adaptation in this situation (14), and current care pathways for people with adult-onset T1D do not systematically address the psychosocial impact of the diagnosis (15).

To address this gap, a team of Danish and UK-based researchers developed a programme through an iterative co-design process involving people with adult-onset T1D (n=31) and healthcare professionals (HCPs) (n=61) (16). This resulted in the “Living with and ADapting to DiabetEs pRogramme (LADDER) aiming at adults ≥ 20 years of age recently diagnosed with T1D. The co-design process highlighted that the programme needed to be phased to accommodate specific needs at different time points, resulting in two distinct complex interventions with several interacting components. First, an intervention consisting of one-to-one sessions soon after diagnosis addressing the impact of the diagnosis on psychosocial health and wellbeing. Secondly, an intervention consisting of group sessions within the first three to 12 months of diagnosis, led by a peer facilitator and an HCP, focussing on how to adapt to a new life with diabetes. A core feature of LADDER is the use of therapeutic communication including active listening, mirroring and open-ended questions. In addition, conversation tools, tailored to facilitate conversations about the psychosocial impact of the diagnosis, are used. Hence, LADDER is a complex intervention characterised by several interacting components and active participant engagement, consequently dependent on the context in which it is delivered (17). To test the feasibility and impact of the two LADDER interventions and determine the most appropriate design for a larger trial, we conducted several pragmatic feasibility studies with a process evaluation in Denmark and the UK (18). To accommodate the differences in the settings, we used different but complementary study designs to explore the acceptability of the intervention and the feasibility of delivering the LADDER intervention in busy diabetes clinics in both countries. A common feature in the study design for each country was the use of a control group. Here, we report the findings of feasibility studies from the UK and Denmark in terms of research processes, delivery of the intervention, the clinical and psychosocial outcomes of LADDER, and suggestions for optimisation.

## Materials and methods

The feasibility studies were designed following the Revised Medical Research Council (MRC) Complex Interventions Evaluation Framework, with an integrated process evaluation (17). In line with the MRC frameworks (17, 18), we adopted a mixed-methods approach to evaluate feasibility, including the various study designs. Quantitative data were used to assess recruitment, retention, data completion, and variability in outcomes, while qualitative data were used to explore the experiences of participants, HCPs and peer facilitators regarding contextual influences, potential benefits of attending the sessions, and perceived barriers and facilitators to delivery.

The data were combined during the interpretation phase through triangulation, allowing for an explanation of quantitative feasibility results and the identification of context-specific mechanisms relevant to the interventions and necessary adjustments. Applying this approach could help guide the optimisation of the intervention and study procedures and reduce uncertainty before deciding how to advance to a full evaluation of the LADDER programme.

The study aims were: 1) to assess the research processes in terms of programme acceptance, recruitment, and completion; 2) to assess the feasibility of delivering the intervention, 3) the feasibility of collecting the psychosocial and clinical outcomes and the potential benefit of participation; 4) to identify key areas for improvement of the intervention content and delivery.

### The LADDER intervention

The biopsychosocial model of adaptation was used as the overarching theoretical framework for LADDER (19, 20), underpinned by psychological and educational theories, to ensure that LADDER optimally addresses the needs of the individual. The theoretical inspiration derives from the following: 1) communication and delivery techniques targeting acceptance, emotions and motivation inspired by acceptance and commitment theory (21), social learning theory (22), and self-determination theory (23). The intervention components were designed to enable the verbalisation of thoughts and feelings related to the diagnosis; normalise and reframe potential challenges and anxieties; and support acceptance, social adjustment, and solution-focused thinking with respect to diabetes self-management.

The LADDER programme consists of two distinct interventions to accommodate different phases of the adaptive process: 1) One-to-one in-person consultations in the diabetes clinic lasting 30-45 minutes one-to-three months after diagnosis with diabetes specialist HCPs trained in facilitating dialogue using conversation tools 1&2 (see **Supplementary Materials 1**–**3**); 2) Group-based psychoeducational sessions (eight hours in total) provided three-12 months postdiagnosis, using conversation tools 2&3 (see **Supplementary Materials 3**–**5**), addressing common psychological and social diabetes challenges; and strategies for managing diabetes in everyday life. The group sessions were held in a neutral space (university meeting room). The sessions used experiential reflective learning techniques to help participants develop and rehearse strategies for navigating these scenarios in a supportive environment (22). The group sessions were facilitated by a peer and an HCP. Peers (a man and a woman living with adult-onset T1D for 11 and eight years, respectively, who were involved in co-designing LADDER) and HCPs (six diabetes specialist nurses, one consultant, one dietician, one psychotherapist) with more than five years of experience with adult-onset T1D care delivered the sessions. In total, the facilitators received six hours of training (for example, therapeutic communication and facilitation skills) prior to the intervention and regular supervision by AF, MDC and JLW while the interventions were ongoing.

The LADDER programme uses three visual conversation tools that assist adults in exploring their experiences following diagnosis to help them process and verbalise the disruption caused by diabetes and make decisions related to their diabetes self-management and self-care. The tools were co-designed by people with adult-onset T1D and diabetes HCPs (16):

Tool 1. The diabetes roadmap – this tool is used soon after diagnosis to help people prepare for their adaptive journey, and it illustrates how adapting to diabetes is an ongoing process. It explores the diagnosis experience and signposts the care pathway people can expect from the healthcare system during the first year after diagnosis. The roadmap is also used to elicit emotional and social responses to the diagnosis that people experience so that these can be expressed and attended to.

Tool 2. Living with diabetes - This tool is used three to 12 months after the diagnosis when people have more experience with diabetes. It illustrates the connectivity between how diabetes affects thought/feelings, the body and everyday life. It encourages people to express and process experiences related to adapting to diabetes, considering not only the biomedical aspects of diabetes but also their thoughts and emotions related to living with diabetes. By highlighting the overlap between these areas, people might get more clarity about what the adaptive process entails and may feel more prepared for different types of experiences. This tool might help people with diabetes see their life with diabetes as a whole and support them in contextualising the demands of diabetes in their lives to foster acceptance.

Tool 3. Adapting to diabetes - this tool was used in the group sessions only. It seeks to normalise a variety of experiences related to adult-onset T1D by using a combination of illustrations depicting common experiences at the time of diagnosis and the ongoing process of adapting to diabetes with quotations related to living with adult-onset T1D. The person with diabetes may recognise themselves in specific examples, i.e., taking insulin at the workplace or having family members comment on their food choice, which might make them feel less alone with their diabetes-related challenges and increase their ability to manage diabetes in different situations.

All three conversation tools and additional tools (24, 25) were used in the group sessions to help participants reflect on common challenges, such as diabetes in the workplace/education, hypoglycaemia, and getting more out of diabetes consultations.

### Study design

The feasibility of the one-to-one consultations and the group sessions were tested independently as they were targeted to different phases of the adaptive trajectory and hence, were aimed at adults at different timepoints following the diagnosis. The one-to-one consultations were tested both in Denmark and the UK. The group sessions were tested in the UK setting but not in the Danish setting. This was due to quality improvement projects in the two Danish settings during the study period involving the development of group-based education for the adult-onset population.

**Figure 1:**
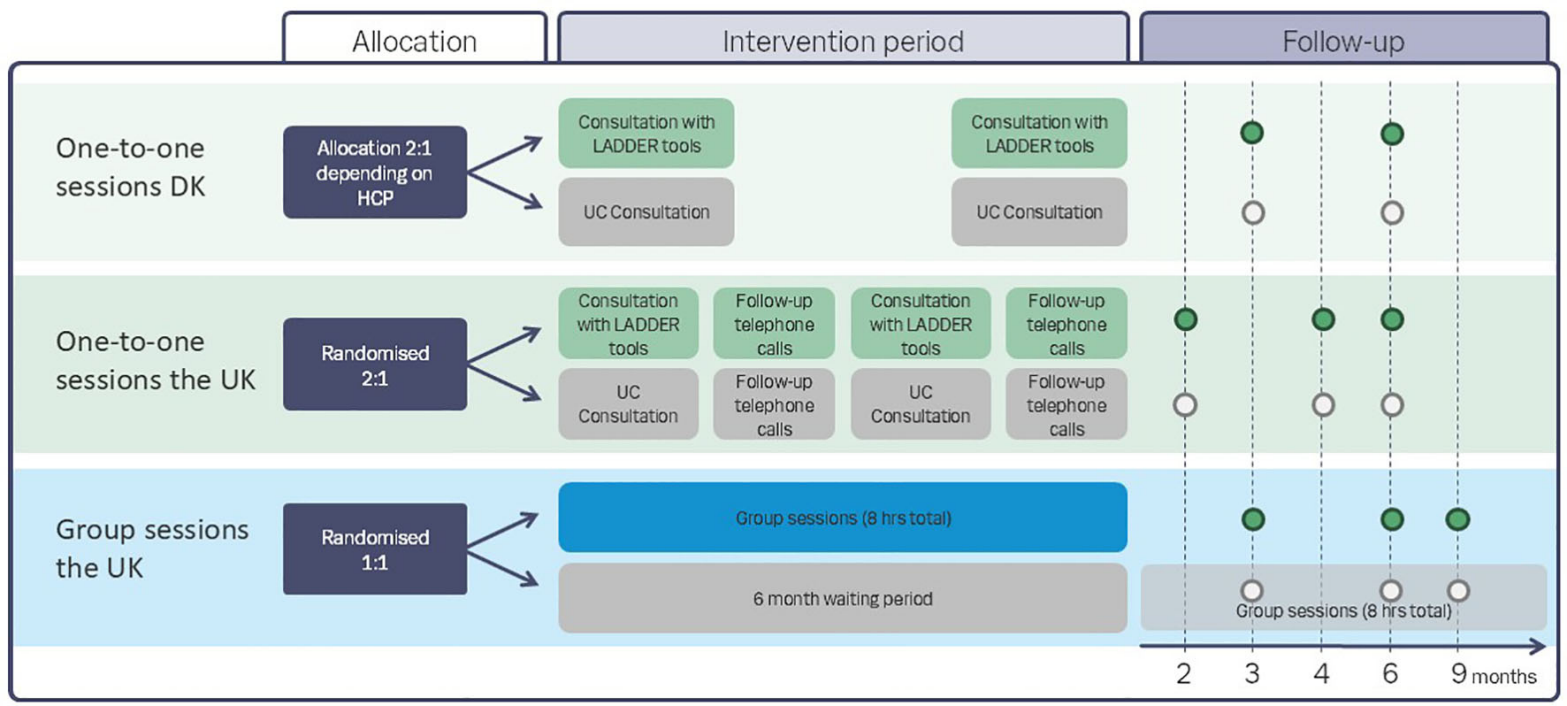
Overview of the intended study designs.

We used similar but different study designs between the two countries to explore the feasibility of applying different designs to a future larger trial. The designs for the one-to-one consultations were as follows: a nonrandomised controlled trial in Denmark and a randomised controlled trial in the UK, and for the group sessions which were only conducted in the UK, a wait-list randomised trial. All trials included a process evaluation. For an overview of the designs, please see **Figure 1**.

#### One-to-one consultations in Denmark

The feasibility study was designed as a nonrandomised controlled trial with a 2:1 allocation to either two one-to-one intervention or usual care (UC) consultations. Allocation depended on whether the HCP they saw at the first study consultation was trained in using the LADDER tools. The first intervention consultation involving the LADDER tools was conducted < three months following diagnosis with the second consultation three to nine months later. UC consultations primarily had a bio-medical focus but also included psycho-social aspects if deemed relevant by the diabetes specialist nurse. UC consultations were delivered by diabetes specialist nurses who were not trained in LADDER. The LADDER consultations replaced a UC consultation in which the tools were used systematically as part of the consultation. Bio-medical or other treatment issues were discussed when relevant. The intervention was delivered by two diabetes specialist nurses, one consultant and one dietician. An evaluation of the consultation was part of the baseline questionnaire for participants (data not presented in this paper), and consequently, participants completed the baseline questionnaire after their first study consultation. Follow-up data were collected three and six months after the second LADDER consultation.

#### Changes to study design (Denmark)

The disruption to clinical work following the COVID-19 lockdown measures introduced major challenges to the recruitment of participants, as clinical procedures were changing, fewer clinical visits were offered, and people with adult-onset T1D were affected by both their new diagnosis and the fear related to COVID-19. To avoid placing additional strain on potential participants immediately after diagnosis, the inclusion criterion related to the time since diagnosis was extended from < three months to nine months postdiagnosis.

#### One-to-one sessions in the United Kingdom

The study was designed as a randomised controlled trial using a preference model for randomisation 2:1 to either an intervention or UC consultation within four weeks after the diagnosis. The second intervention consultation was held three to nine months later. As in Denmark, the LADDER consultations replaced a UC consultation. In the UK, a follow-up telephone consultation was added two weeks after each LADDER consultation to follow up on any questions. This feature was added to the UK intervention, as the frequency of face-to-face appointments was lower in the UK setting. The intervention was delivered by diabetes specialist nurses, whereas UC consultations were delivered by diabetes specialist nurses and a dietician. The participants completed the baseline questionnaire before their first study visit. Follow-up data were collected two, four, and six months after the second LADDER consultation.

#### Changes to study design (UK)

Owing to challenges in getting in touch with potential participants within the first four weeks postdiagnosis, eligibility was changed to eight weeks postdiagnosis.

#### Group sessions

The feasibility study testing LADDER group sessions was conducted in the UK with a randomised wait-list design involving adults who had been diagnosed three-12 months prior to consenting to participate in the study. Group sessions were offered either as weekly evening sessions over the course of four weeks lasting two hours or two sessions lasting four hours each either in the afternoon or on a Saturday. The participants completed the baseline questionnaire before attending the first session. Follow-up data were collected three, six, and nine months after the end of the last group session.

#### Changes to the study design

Owing to a lower-than-expected recruitment rate to the group sessions at the start of the study, we had to abandon the wait-list design. Eligibility criteria were increased to 18 months postdiagnosis because of the challenges of getting in touch with potential participants and their difficulties attending the planned sessions due to scheduling issues.

### Participants and settings

The primary eligibility criterion for recruitment to the study in both countries was a diagnosis of T1D in adulthood and aged ≥20 years. The exclusion criteria were severe physical/mental illness; pregnancy; acute unstable retinopathy; significant learning difficulties; or inability to speak or understand English or Danish. The eligibility criteria for taking part in the one-to-one consultations and the group sessions were different. The revised eligibility criteria for recruitment to one-to-one consultations was a recent diagnosis of T1D (< nine months in Denmark, < eight weeks in the UK), and three to 18 months postdiagnosis for the group sessions. Participants were only eligible for one of the LADDER interventions. Initially, an exclusion criterion for the group sessions was current or planned attendance at other structured education programmes, such as the Dose Adjustment for Normal Eating (DAFNE) programme. However, this exclusion criterion was abandoned, as two participants were offered and attended DAFNE while waiting to attend the LADDER sessions.

Participants in Denmark were recruited from two diabetes centres, one in the Capital Region (Steno Diabetes Center Copenhagen) and one in the Region of Southern Denmark (Steno Diabetes Center Odense) between 28^th^ January 2021, and 27^th^ May 2022. Collectively, the Danish centres have approximately 140 cases of adult-onset T1D per year.

In the UK, participants were recruited from three diabetes centres in South London; Guy’s and St Thomas’ Hospital, King’s College Hospital (one-to-one consultations or group sessions) and Lewisham and Greenwich Hospital (group sessions) between May 16^th^, 2022, and July 7^th^, 2023. Collectively, these centres have 80–100 cases of adult-onset T1D per year.

All participating centres, except Lewisham and Greenwich Hospital, were involved in developing the LADDER programme.

Potential participants for the one-to-one consultations were screened and approached about taking part in the study by a clinician in their diabetes clinic. Those interested in participating in the study received verbal and written information from the research team. Potential participants for the group sessions were identified from clinical lists by the clinicians and were invited to take part in the study via email or letter. The UK participants receiving one-to-one sessions were asked about their willingness to be randomised to either the intervention group or the UC group. The participants who received the group sessions were asked about their willingness to be randomised to either the intervention or wait-list group. Participants who agreed to be randomised were randomised to either the intervention group or the UC (one-to-one consultations) or wait-list group (group sessions) through a computer-generated randomisation programme in blocks of 10 by members of the study team. All participants provided written informed consent.

### Feasibility measures

1) Research processes: To assess the feasibility of the research processes, we measured the recruitment and retention rates and randomisation acceptability (the UK).
2) Delivery of the intervention: We explored acceptability of delivering the intervention through semi-structured interviews with study participants and HCPs (DK and UK) and peer facilitators (UK).
3) Clinical and psychosocial outcomes: We assessed data completion for the clinical and psychosocial outcomes. The clinical outcomes of interest were HbA_1c_, attendance at diabetes appointments, hypoglycaemia, and severe hypoglycaemic events (for which third-party assistance is required). The psychosocial impact was measured via questionnaires that intended to model the interventions’ biopsychosocial targets: psychological well-being (WHO-5 General Well-being) (26), illness perception (Brief Illness Perception Questionnaire) (27), impact of diabetes (DAWN2 Impact of Diabetes Profile) (28, 29), support for diabetes self-management (DAWN2 Support Profile) (28, 29), general stress (Perceived Stress Scale) (30), autonomy support from health care professionals (Health Care Climate Questionnaire) (31), attachment (Experience of Close Relationships) (32), diabetes resilience (Diabetes Empowerment Scale-Short Form) (33), diabetes distress (Problem Areas In Diabetes) (34), and illness identity, with four subscales: acceptance, rejection, engulfment, and enrichment (Illness Identity Questionnaire) (35). The questionnaire consisted of in total 99 items about the psychosocial impact and took approximately 20 minutes to complete. The participants in the one-to-one consultations had limited experience of living with diabetes at baseline; hence, questionnaires about diabetes distress, diabetes resilience, and illness identity were not completed at baseline but rather in all follow-up questionnaires.
4) Optimisation: Suggestions from participants regarding changes in the interventions and delivery were collected through semi-structured interviews.

### Data collection and analysis

We used a multimethod approach to collect and analyse data, as described below, to capture a variety of perspectives regarding the feasibility studies.

1) Research processes: Data were collected from data logs completed during the study. Data are presented descriptively as rates for consent and programme completion, and number of participants who agreed to be randomised for the one-to-one consultations in the UK. We defined completion as having attended two one-to-one consultations, and for the group sessions, we defined completion as having attended three out of four weekly sessions or one out of two weekend sessions.
2) Delivery of the intervention: The semi-structured interviews were conducted either in person, on the phone or via Microsoft teams within four months of the last consultation/group session by MDC and ER in Denmark and by MDC and REH in the UK. The interviews were audio-recorded and transcribed verbatim, anonymised, and then organised in NVIVO 12 for analysis (36). Regarding the feasibility of delivering the intervention, we used a deductive approach to explore the acceptability of the intervention (whether LADDER is agreeable, palatable, and satisfactory), appropriateness (the perceived fit, relevance or compatibility of LADDER to address psychosocial adaptation in people with adult-onset T1D), and feasibility (the extent to which LADDER could be successfully carried out in the study settings).
3) Clinical and psychosocial outcomes: HbA_1c_ values, attendance at diabetes appointments, hypoglycaemia and severe hypoglycaemic events were collected retrospectively from the participants’ electronic records by the clinicians when available. The recorded HbA_1c_ level closest to the completion of the study questionnaires was used. Data on psychosocial outcomes were collected from questionnaires completed online. In Denmark, participants received a collated questionnaire via a secure platform (e-boks), which they actively had to log on to retrieve the questionnaire. Their responses were stored in REDCAP. In the UK, participants were emailed a link to the questionnaire via Online Survey. Descriptive statistics were used to report baseline data. Furthermore, the psychosocial outcomes were investigated via semi-structured interviews exploring the perceived impact of participating in the interventions. The interviews were conducted and transcribed as described above. The interview data were analysed with inspiration from the six steps of reflexive thematic analysis (37). Initially, all transcripts were read through several times to obtain a sense of the content; they were then coded according to the participants’ responses in relation to their experience with LADDER. The codes were then used to identify patterns and meanings expressed in statements related to adapting to a life with diabetes. Statements were then compared to identify differences and similarities and to develop themes.
4) Optimisation: Questions about optimisation were asked as part of the semi-structured interviews described above. In addition, in the UK, information regarding optimisation was collected at a final study event involving study participants, the advisory board, HCPs and peer facilitators. Data were analysed deductively by grouping suggestions for optimisation according to the programme structure, content, delivery, and outcomes.

### Sample size

The sample size was estimated based on consensus guidance for feasibility evaluations, the aim of which is to present the data required (standard deviation of outcomes) to formulate a power calculation for a larger trial (38). Following this guidance, for the one-to-one consultations, we aimed to recruit 50 participants from Denmark and 26 participants from the UK, and for the group sessions, we aimed to recruit 40 participants (UK only). The statistical analyses were conducted independently for each country and separately for the one-to-one consultations and the group sessions.

### Ethics approval and consent to participate

In Denmark, the permission to conduct the study was obtained via the Capital Region by the Danish Data Protection Agency (reference P-2020-1187). The UK study received ethical approval from North-West Preston REC (reference 22/NW/0053). The studies complied with the Helsinki Declaration, and all participants provided written informed consent before participation.

## Results

The results are presented for each country and each intervention in relation to the feasibility of 1) the research processes, 2) the delivery of the intervention, 3) clinical and psychosocial outcomes, and 4) optimisation. Data are presented together for the clinical and psychosocial outcomes and suggestions for optimisation. The observational data and interview data are integrated where appropriate.

### Research processes

#### One-to-one sessions

**Figure 2:**
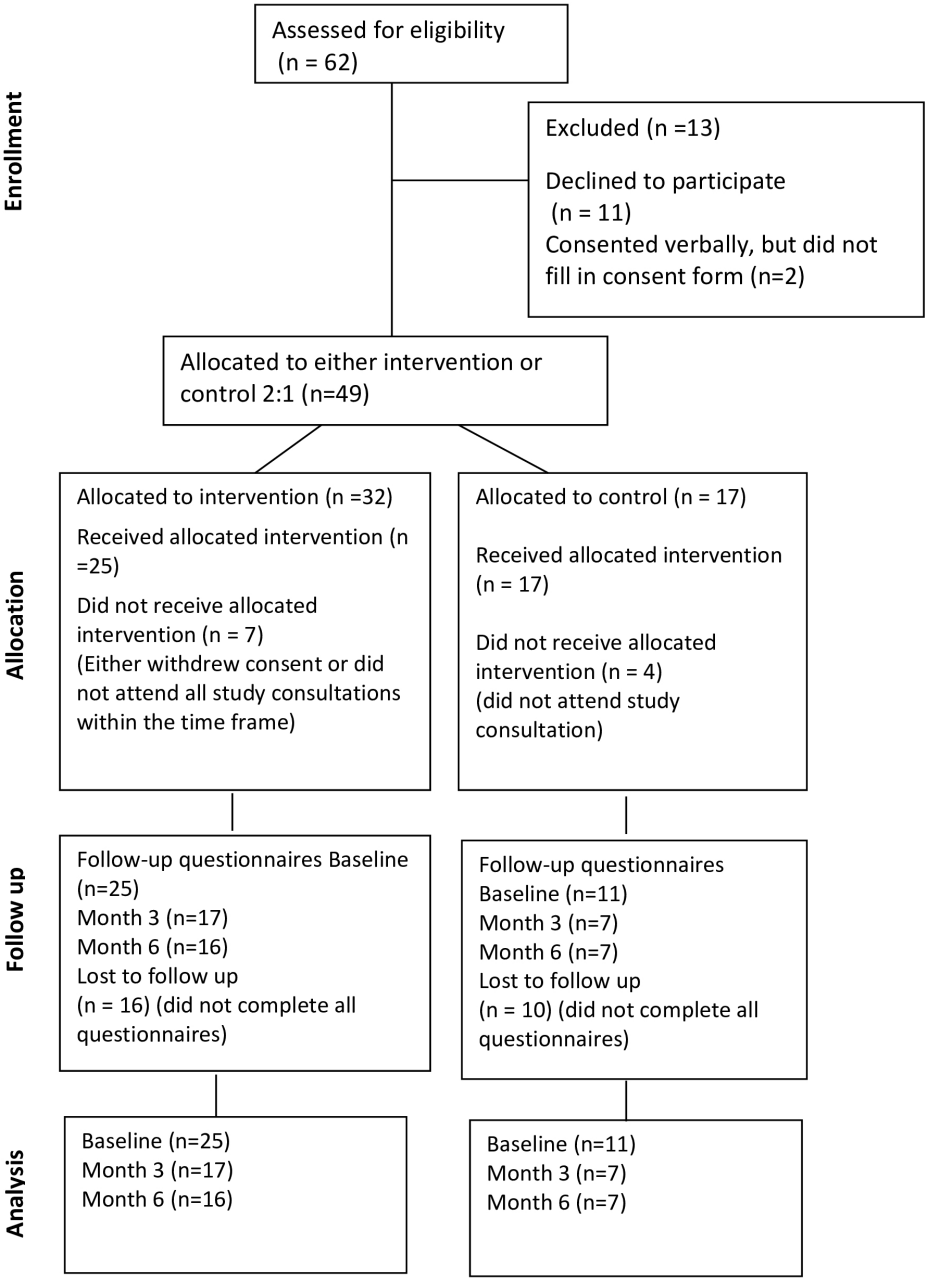
Consort diagram of one-to-one session in Denmark.

Denmark: Sixty-two eligible adults were invited to participate in the study, and 51 agreed, of whom 49 (79%) completed the consent form (for participant characteristics, please see **Table 1**). Among the reasons for not consenting were feeling overwhelmed by the diagnosis, not wanting to complete questionnaires and generally dislike of taking part in studies. Among the 49 people who consented, 38 received the intervention. Thirty-two participants were allocated to the intervention group, and 17 were allocated to the UC group. Of those in the intervention group, 25 received the intervention, and seven did not receive the intervention. In the control group, 13 out of 17 received UC (either withdrew their consent at the time of their first consultation or did not attend all consultations within the study period), resulting in a retention rate of 78%. For an overview, please see **Figure 2** Consort diagram of one-to-one consultations in Denmark.

**Table 1:** Participant characteristics.

Participant characteristics	One-to-one sessions				Group sessions	
	The UK		Denmark		The UK	
Female	7	(58%)	18	(50%)	10	(50%)
Male	5	(42%)	18	(50%)	10	(50%)
Age (years- median/IQR)	29	(8.8)	43.6	(23.6)	31	(7.8)
Relationship Status (n, %)						
Single	4	(33%)	10	(28%)	6	(30%)
Married/Partner	8	(67%)	26	(72%)	13	(65%)
Divorced	0	(0%)	0	(0%)	1	(5%)
Living Status (n, %)						
Alone	0	(0%)	7	(19%)	9	(45%)
Partner	3	(29%)	26	(72%)	7	(35%)
Family	8	(71%)	1	(3%)	4	(20%)
Others	0		2	(6%)	0	
Ethnicity (n, %)						
Black	0	(0%)	NI		1	(5%)
Asian	0	(17%)	NI		1	(5%)
White	10	(83%)	NI		18	(90%)
Other	2	(0%)	NI		0	(0%)
Education (n, %)						
Secondary	3	(25%)	5	(14%)	2	(10%)
Higher education	9	(75%)	31	(86%)	18	(90%)
Employment (n, %)						
Working	12	(100%)	NI		18	(90%)
Unemployed	0	(0%)	NI		0	(0%)
Retired	0	(0%)	NI		0	(0%)
Student	0	(0%)	NI		2	(10%)

NI, Information not available.

**Figure 3:**
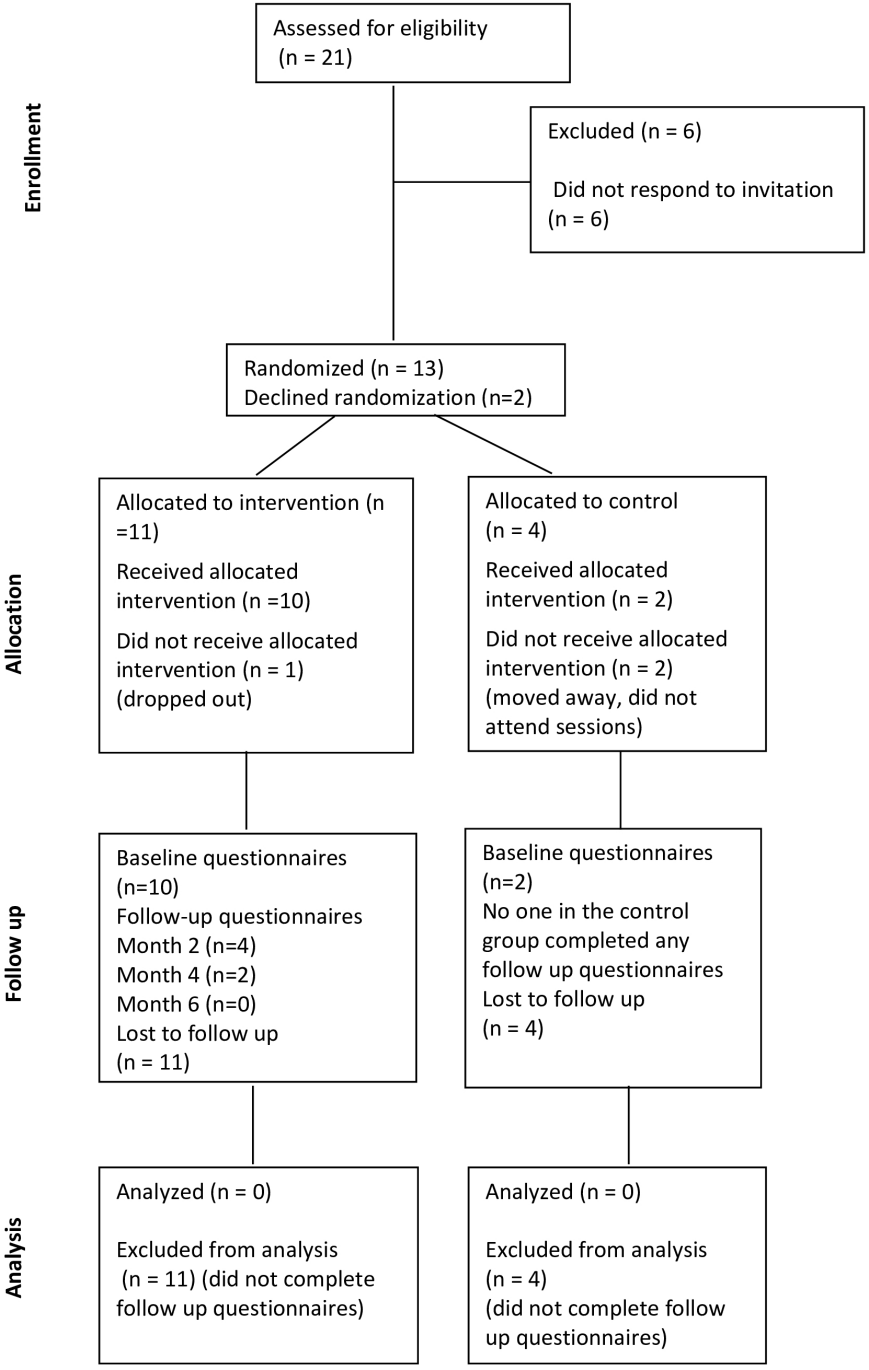
Consort diagram of one-to-one session in the UK.

The UK: Twenty-one eligible adults were invited to participate, and 15 people consented (71%) (for participant characteristics, please see **Table 1**). Thirteen participants consented to randomisation. Eleven participants were allocated to the intervention group, and four participants were allocated to the UC (active control) group. Two participants declined randomisation and were offered their preference allocation to the intervention sessions. Of the 15 people who consented, 12 received the intervention, and three did not receive the intervention (either did not complete the baseline questionnaire/withdrew their consent at the time of the first consultation or did not attend all consultations within the study period), resulting in a retention rate of 80%. For an overview, please see **Figure 3** Consort diagram of one-to-one consultations in the UK.

#### Group sessions

**Figure 4:**
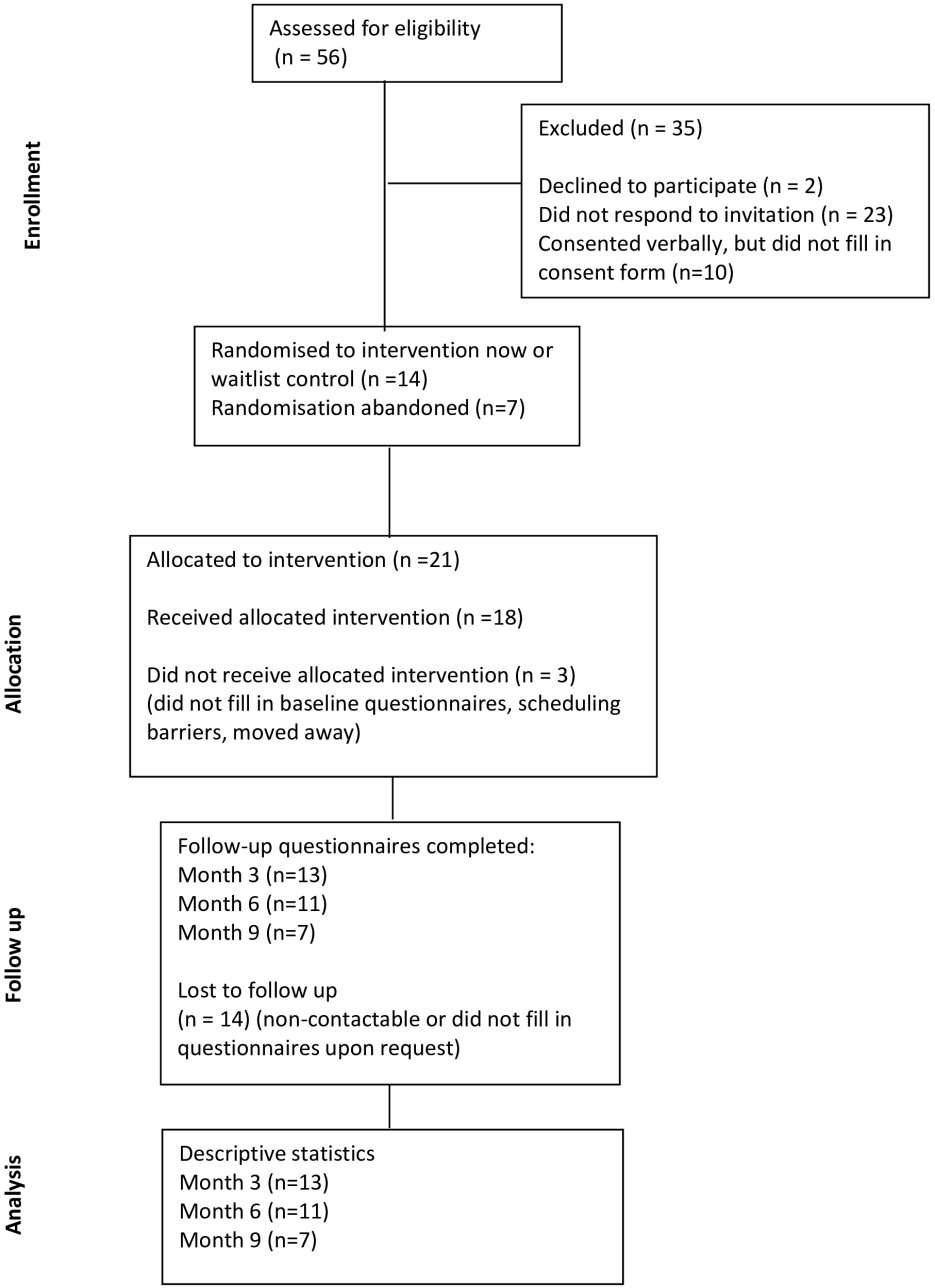
Consort diagram of group sessions in the UK.

The clinicians identified 56 participants who were eligible. All of them received information about the study with an invitation to hear more from their clinicians in person or via letter or email. Two participants declined the invitation, and 23 did not respond. Thirty-one people verbally consented to be contacted about participation. Twenty-seven participants verbally consented to participate, only 21 of whom completed the electronic consent form (recruitment rate 37%). The main reason for not consenting was being unsure of availability to attend the sessions. The initial 14 participants were randomised to the intervention or wait-list control group. However, it took longer than expected to recruit participants and fill the groups. Consequently, maintaining a randomised wait-list design was not feasible; instead, participants were allocated to the group sessions on a rolling basis. Eighteen out of the 21 people who were recruited completed the full intervention (retention rate 85%). Of the three people who did not proceed with completing the questionnaires and attending the sessions, two faced scheduling barriers, and one withdrew after one session (participant characteristics in **Table 1**). Four groups with four to six participants in each were carried out. For an overview, please see **Figure 4** Consort diagram of group sessions in the UK.

#### Delivery of the intervention

In Denmark, we conducted semi-structured individual interviews with 18 intervention participants and five HCPs (three diabetes nurse specialists, one dietician, one consultant), and in the UK five intervention participants, and three HCPs (all diabetes specialist nurses) were interviewed to evaluate the feasibility of delivering one-to-one consultations.

For the group sessions, we interviewed eight participants individually, two peer facilitators and one HCP (psychotherapist). Most of those who were interviewed reported that the intervention was acceptable and appropriate for them. Key features that made both interventions acceptable and appropriate were the provision of a safe space to reflect on their feelings about the diagnosis and their adaptive process, including the normalisation of experiences as illustrated by visual tools. For the group sessions, participants particularly highlighted that the opportunity to meet peers, sharing experiences and identifying with others added to the relevance of the programme (see **Table 2** for supporting quotes).

**Table 2:** Supporting quotations regarding the feasibility of delivering the LADDER interventions.

Implementation indicator	Quote from participant	Participant
Acceptability	I think it was very interesting and definitely beneficial in terms of talking about some of my feelings or some thoughts that popped up since I was just diagnosed.	4A UK*
Acceptability	I think it was quite nice to have something (the tools) to lean on that you could talk from.	111A Denmark
Appropriateness	I thought it was a really nice practical approach because I like to be yeah bad things might happen but at least I feel equipped. I think that’s what I felt, I feel that was a really positive way to not push it under the rug, to be like oh you know what there are things that you can do	11B UK
Appropriateness	It is some kind of tool that all of us as newly diagnosed diabetics can relate to. (…) you think ‘well, someone has thought about this’. Someone has thought about how we're going to learn to live with this.	207A Denmark
Feasibility	I think it is really really good, and it also makes something in my teaching different, because we get to talk more about ‘how you are’ with the patient and ‘what do you feel’ and ‘what have you experienced’ rather than you did this and that. So, we include more of the social and emotional aspects of the same topic.	HCP Denmark
Feasibility	So, it gives them permission just to share and they might not have shared it with anyone, or it could be linked to – and that allows the healthcare professional, because they're not doing the talking, they're just listening, to then think ask, be curious.	HCP UK

*Participant ID, Intervention (A, one-to-one sessions; B, group sessions); Country.

The participants who were interviewed expressed that their decision-making in terms of taking part in the study were influenced by how they felt about their diabetes diagnosis, the way they were approached by their clinician and the support they received. Furthermore, the setting and structure of the intervention, including the duration and timing of the sessions, were practical issues that influenced their ability to take part. The HCPs who delivered LADDER highlighted the benefit they had gained from the training they had received prior to the start of the study. They pointed to the need for HCPs to be upskilled to facilitate the programme in other settings with adequate planning around training and delivery of the intervention and being part of a team with opportunities for supervision.

### Clinical and psychosocial outcomes

In the Danish study 36 people (25 in the intervention group and 11 in the UC group) provided baseline data (73%). The follow-up data were completed by 24 participants (17 and seven, respectively) (49%) at three months and by 23 (16 and seven, respectively) (47%) at six months. Regarding the one-to-one consultations in the UK 12 people (10 in the intervention group and two in the UC group) provided baseline data (80%). Four participants provided follow-up data (27%) at two months, two at four months, and 0 at the six-month follow-up. The outcome data relating to the group sessions were completed by 20 participants at baseline (95%). The follow-up questionnaires were completed by 13 (62%) people at three months, 11 (52%) at six months, and seven (33%) at nine months. We could not obtain all the quantitative data we had planned for. Data on some of the clinical outcomes, such as attendance at diabetes appointments, hypoglycaemia, and severe hypoglycaemic events (for which third-party assistance is required), were not collected due to logistical barriers. The research team did not have ethical approval to access records directly and were dependent on busy clinical staff to provide data from different record keeping systems that were time consuming to collect.

HbA_1c_ data are available for the Danish study only because it was not possible to access the HbA_1c_ data in the UK study. Due to the low number of participants, the low response rate at follow-up, and the lack of relevant control groups no statistical comparisons were made.

The questionnaire data is presented descriptively for each country in **Tables 3**–**6**.

**Table 3:** Participant outcome measures regarding one-to-one consultations in Denmark.

Questionnaire	Baseline (n=36)		3 m (n=24)		6 m (n=23)	
	Intervention (n=25)	Control (n=11)	Intervention (n=17)	Control (n=7)	Intervention (n=16)	Control (n=7)
WHO-5-General Well-being	60 (22)	52 (26)	NA	NA	68 (30)	60 (12)
Perceived Stress	12 (5.5)	14 (6.5)	14 (7.5)	18 (12)	17 (9.2)	19 (4)
Brief Illness Perception	40 (17,5)	38 (4.5)	29 (14)	37 (15)	28 (21.8)	30 (18)
DAWN2 Impact of Diabetes Profile	67 (13)	62 (12)	64 (8)	67 (12)	68 (14)	67 (7)
DAWN2 Support Profile	73 (28)	73 (33)	80 (32)	73 (46)	46 (20)	40 (7)
Health Care Climate	6.8 (1)	5.8 (1.4)	NA	NA	NA	NA
Experience of Close Relationships	3 (1)	3.5 (3)	NA	NA	NA	NA
Problem Areas in Diabetes	NA	NA	28.8 (20.6)	33.8 (47.5)	29.4 (21.2)	33.8 (33.8)
Diabetes Empowerment	NA	NA	2.4 (0.6)	2.4 (0.7)	2.5 (0.9)	2.3 (1.1)
Ilness Identity Subscales						
Illness Identity Acceptance	NA	NA	3.5 (1)	3.5 (1)	4 (1.5)	4 (0.5)
Illness Identity Rejection	NA	NA	3 (0.8)	2.5 (1)	2.5 (1)	2.5 (1)
Illness Identity Engulfment	NA	NA	3 (1.8)	2.5 (1)	2.5 (1)	3 (0.5)
Illness Identity Enrichment	NA	NA	3 (1.2)	3 (1)	3 (1.9)	2.5 (1.5)

**Table 4:** HbA_1c_ outcomes regarding the one-to-one consultations in Denmark.

Outcome	Baseline (n=39)		3 m (n=31)		6 m (n=27)	
	Intervention (n=29)	Control (n=10)	Intervention (n=25)	Control (n=6)	Intervention (n=21)	Control (n=6)
HbA_1c_ (mmol/mol)	108 (33.5)	96 (31.3)	50 (14.5)	53 (18.7)	54 (15)	54 (25.8)

**Table 5:** Participant outcome measures regarding the one-to-one consultations in the UK.

Questionnaire	Baseline (n=12)
WHO-5-General Well-being	56 (41.5)
Perceived Stress	24 (4.8)
Brief Illness Perception	56 (13)
DAWN2 Impact of Diabetes Profile	44 (15.1)
DAWN2 Support Profile	38 (9.1)
Health Care Climate	5.6 (1.9)
Experience of Close Relationships	4.5 (0.8)

**Table 6:** Participant outcome measures regarding group sessions in the UK.

Questionnaire	Baseline (n=20)	3 months (n=13)	6 months (n=11)	9 months (n=7)
WHO-5-General well-being	58 (26)	60 (25)	64 (16)	68 (36)
Perceived Stress	21.5 (5.8)	21.0 (3.0)	21.0 (6.0)	22.0 (7.0)
Brief Illness Perception	56 (10)	55 (7.3)	59 (11)	59 (23)
DAWN2 Impact of Diabetes Profile	45.2 (17.2)	45.2 (14.3)	45.2 (20.6)	52.4 (11.1)
DAWN2 Support Profile	37.4 (11.5)	33.7 (9.5)	38.1 (14.2)	38.1 (11.8)
Health Care Climate	5.6 (2.2)	4.8 (1.2)	4.8 (1.1)	6.0 (4.3)
Experience of Close Relationships	3.4 (0.9)	NA	3.4 (0.9)	NA
Problem Areas in Diabetes	29.2 (35.3)	29.4 (24.5)	33.9 (24.2)	16.8 (17.8)
Diabetes Empowerment	3.6 (0.6)	3.6 (0.8)	4.0 (1.1)	3.9 (0.4)
Illness Identity subscales				
Illness Identity Acceptance	3.8 (0.9)	3.7 (1.3)	3.8 (0.8)	3.8 (0.6)
Illness Identity Rejection	2.2 (1.3)	2.2 (1.2)	1.8 (1.4)	1.8 (1.0)
Illness Identity Engulfment	2.6 (1.6)	2.6 (1)	2.6 (0.8)	2.1 (0.8)
Illness Identity Enrichment	3.6 (1.7)	3.6 (0.9)	3.9 (1)	3.9 (1.5)

The HbA_1c_ results are shown in a separate table because data were available from more participants (**Table 4**).

We analysed the qualitative data by combining data on the experienced benefits across one-to-one consultations and the group sessions from both countries. Our analysis identified two overarching themes, and one subtheme related to the potential ability of LADDER to promote early positive adaptation to life with T1D by enhancing psychological and social well-being and possibly prevent the development of diabetes distress. The themes were as follows: 1) A safe space to process the diagnosis experience with subtheme 1a: the benefit of peer support, and 2) Navigating the new normal.

The following sections outline the participants’ experiences with the potential benefits of attending LADDER. Quotations from participants are integrated in italics with their study ID and intervention allocation (A: one-to-one sessions, B: group sessions) in brackets following their quote.

#### Theme 1: A safe space to process the diagnosis experience

The participants reported that the LADDER interventions provided a safe space to share their thoughts and feelings about their life with diabetes. The discussions supported the participants in developing language about their diabetes, normalised and acknowledged their experiences and helped them reflect on their situation and support needs. While there were similarities between the experiences of participants in both interventions, the subtheme *the benefit of peer support* details the specific experiences of those attending the group sessions.

Most participants had very little experience with clinical settings and interactions with HCPs before their diagnosis. In particular, the participants in the one-to-one sessions expressed that the LADDER consultations made them *“more comfortable in the physical environment of the hospital” (5A).* The opportunity to have close, regular contact with nominated HCPs helped participants establish good rapport and build relationships with them. According to the participants in both interventions, the LADDER sessions created a safe space for reflecting on their experiences related to diabetes. The visual tools, which illustrate common experiences of the adaptive process, could reveal hidden and unrecognised emotions in a supportive way by helping participants find a language for their diabetes experience. The conversation tools encouraged participants to speak about topics that they might not have considered or recognised the impact it had on them. For example, one participant said, “*It gave me the opportunity to go through some of the things that I hadn’t necessarily thought about, or to be able to verbalise some of the things that were a bit difficult to put into words” (220A).*

Recognition of their thoughts and feelings could provide other perspectives on their situation and experiences, and participants were *“able to take away a better perspective with my condition” (5B).* Some participants also highlighted that the recognition developed through the LADDER consultations *“helped me understand myself and my diabetes and my reactions to change” (3A).*

Our findings indicated that the focus on the needs and experiences of the participants created a different flow in the conversations compared to their previous consultations without the LADDER tools. The LADDER tools prompted the participants to share their personal situation in more detail as they found the discussion relevant to their needs. The signposting of present and potential future emotional reactions, such as feeling overwhelmed by managing diabetes, was perceived by some to be helpful in preventing experiences of additional distress. The participants felt more prepared and able to voice their concerns and take measures to avoid overwhelming negative reactions. For example, one participant said, “*I thought it was a really nice practical approach because bad things might happen, but at least I feel equipped. I think that’s what I felt; I feel that was a really positive way to not push it under the rug” (10B).*

The participants expressed that the visual conversation tools supported them in processing their experience: *“You can talk your way through the process, coming to some kind of realisation that now you are diabetic and have a chronic disease and things are different than they were before” (224A).*

They reported that the LADDER conversations encouraged reflections on emotional reactions to the diagnosis, as the tools highlighted that diabetes affects different aspects of people’s lives. Experiencing such conversations set a guiding principle for future consultations regarding the emotional side of adapting to diabetes as expressed by one of the participants: “*It feels like you just went a little bit deeper [in the conversation] we talked about a whole bunch of different perspectives on how I really felt and thought about it without really having put it into words before, so I really appreciated that. I walked away feeling almost relieved, like a weight had been lifted from my shoulders, and it was really really great” (103A).*

Our findings suggested that the openness and honesty accommodated by the LADDER conversations legitimised a variety of emotions and could reassure participants that it is normal to have such feelings and provided hope for managing diabetes in the long term.

*“It [knowing that others with T1D feel the same way] just made me feel like it’s OK, like I can get through it, and I know there’s going to be good times, there’s going to be bad times, but I mean I can get through it, everyone has” (2A).*

The recognition of the common experiences of the diagnosis and life with T1D reflected in the visual conversation tools and the discussions in the group sessions could make participants feel less alone with their condition, as highlighted by this participant: *“suddenly you feel like you’re not all by yourself anymore” (204A).*

The central role of the relationship with HCPs in participants’ experience of their new life with diabetes was highlighted, as the participants revealed that they felt seen and heard as the HCPs explored, validated, and normalised their reactions. The HCPs posed open-ended questions, acknowledged their experiences, and provided time for reflection. Having space to reflect on their own situation could help to establish trust among the person with diabetes and HCPs, and lead to open and honest conversations about what having diabetes meant to them.

#### Subtheme 1.a: The benefit of peer support

The participants in the group sessions emphasised the added value of being in the same room as people with similar experiences. Having someone more experienced with life with T1D as a facilitator was an important driver of providing trust within the group very early in the sessions as well as providing hope for their ability to manage diabetes going forward. Peer support promoted a deeper sense of being understood by likeminded people. *“I just took away that lots of other people are in the same boat as you are, that you’re not alone, and actually, it’s just like the things that you experience are just so common” (10B).* The connection and trust in the group was formed very quickly, allowing participants to relate honestly to each other’s experiences and approaches to life with diabetes, as highlighted by this participant: *“I learned how different people do things. I got to see how other people deal with what I’m dealing with” (1B).*

The group setting allowed participants to articulate and mirror their emotional experiences with one another, which could help them normalise and put their experiences and emotional responses into perspective, as explained by this participant: *“Being able to kind of vent frustrations, that each other would have a better understanding of, you know, like the sort of silly things that someone’s relative might say that they think is helpful but is, really pissing other people off. Having a sort of context of people who understand the* sp*ecifics of you that might upset someone is really useful” (7B).*

#### Theme 2: Navigating the new normal

The participants expressed that they felt overwhelmed by their new life situation. They needed to gain perspective on their life with diabetes by knowing what was going to happen with their care and their everyday lives. There was also a need to understand that it takes time to adjust to living with diabetes by recognising the complexity and demands of their new situation.

Our findings suggest that the LADDER one-to-one conversations involving the diabetes road map tool could assist in eliminating some of the confusion participants experienced, as it outlined the adaptive process in a structured way, explaining common phases that people go through. This could make the participants feel reassured that there was a plan for their care and offer them a view on their current situation from a different perspective. The participants appreciated the overview provided to them. The roadmap also revealed what kind of support HCPs could offer, making it clearer for participants what to expect from their diabetes team which could inform them about how to navigate the healthcare system and find the support they needed.

By highlighting the phasic nature of the journey into a life with diabetes, participants indicated that the LADDER sessions helped them feel less under pressure to get everything right in a short space of time. Our findings suggest that the awareness of the nonlinear adaptive process might enable the participants to be better prepared for and accept fluctuations in their life with diabetes. This could allow participants to have a more realistic view of their diabetes management. Sharing their thoughts and expectations reassured them that they would eventually learn to live with diabetes, as indicated by a participant in the following way: *“I’m on a continuum where it fluctuates like anything else [ … ] it’s like the rest of your life essentially. So, there’s time for improvement, and it’s going to be gradual rather than everything all at once” (3A).*

Following the diagnosis, the participants had to learn to live with diabetes and find their own way of integrating diabetes into their everyday lives. While that required knowledge about managing diabetes and insight into how to seek help when needed, participants also had to allow for this to be a process of realising the impact diabetes had on their lives. The participants indicated that the LADDER sessions supported them in finding their *“own way of balance for how I treat it and see it” (5B)* and *“feel a bit more like I know what I’m doing” (6B)*. As participants tried to find ways to manage diabetes in different situations, the LADDER discussions could support them in acknowledging and reflecting on the complexity of managing diabetes as identified by this participant: “*It [the LADDER sessions] really illustrated the uncertainty surrounding everyday life in a good way. Where previously it’s been difficult to put it into words exactly although it’s been in the back of my mind all the time” (220A).*

Similarly, participants stressed that the LADDER sessions could normalise the burden and unpredictability of managing diabetes 24/7 in a way that helped them show more compassion towards themselves by not being *“so hard on myself”* (5B). Previously, some participants had *“a constant fixation with perfection”* (2A) or felt *“like a failure”* (5B) and were able to relate to their feelings and expectations around diabetes care in a more flexible way. For example, one participant said, *“[You realise] that you do have these ups and downs and days where it just does not work no matter how hard you try and just kind of give yourself a break from that”* (9A).

As participants were recently diagnosed with T1D, they were trying to find a space in their life for diabetes. They needed to join the dots of the different ways in which diabetes affected their personal lives. While participants were in the process of developing competences in managing diabetes, they experienced a flux in terms of their sense of influence over their life situation. Our findings highlighted how participants felt that the LADDER sessions supported them in getting a broader sense of what life with diabetes entailed. They expressed that the “Living with diabetes” tool could provide a new way of recognising and reflecting on how the physical management of diabetes was linked together with psychological and social aspects: *“It made it easier for me to put into perspective, what I am going through right now, and put into words how I really feel my everyday life is”* (103A).

The participants reported that living with diabetes affected their social relationships in different ways and that the LADDER sessions supported them in having open and honest conversations with their partner, friends, and work colleagues, with some even using the visual tools as a conversation starter to help explain their experiences. Having expressed their reflections during the LADDER sessions, some participants were able to explain the impact of diabetes to others in a more confident way. As an example of what had changed, a participant said, *“I had a career change, and I approached that with more openness around my diabetes than I did at my previous job. [LADDER] probably helped me be more open and less shameful about [diabetes management]”* (10B). Challenges in their relationships due to their new situation, such as other people’s reactions to the diagnosis or lack of understanding, were also discussed in the LADDER sessions. This could help participants set boundaries and ask for support by involving others in diabetes management to the extent that they felt comfortable.

The participants relayed that the reflections facilitated by the LADDER sessions were helpful in the ongoing process of navigating their new situation and finding space for diabetes in their lives. For example, a participant explained that they had gained a new perspective on diabetes:

*“I feel a lot more like I just have it, it’s a part of me [ … ] there are days where I just kind of hate it, but those are fewer than they were, which is good!”* (6B), and another participant shared their reflection on diabetes: *“I think [it] made me realise that it doesn’t have to take over your whole life, it is just part of it”* (11B).

#### Suggestions for optimisation

The participants suggested that the LADDER programme should be integrated into the wider care pathway for people with adult-onset T1D. They felt that one-to-one consultations and group sessions could serve as practical ways to support both people with T1D and HCPs in addressing short-term, mid-term and long-term diabetes-specific challenges and management issues. For example, those who attended the group sessions thought that some sessions could replace individual appointments in the diabetes clinic, as relevant topics could be covered more efficiently and information could be shared more widely, particularly when HCPs may not have time to go more in-depth during individual appointments. The participants had several suggestions to optimise the LADDER interventions. The suggestions were not completely aligned among participants, i.e., regarding the number of participants in the group sessions; however, the most common suggestions are outlined in **Table 7** and grouped according to the structure, content, delivery, and outcomes.

**Table 7:** Suggestions from participants attending either one-to-one consultations or group session for the optimisation of LADDER.

Item	One-to-one sessions	Group sessions
Structure	- Offer the first LADDER consultation four-to-six weeks postdiagnosis (Danish participants) - Possibility to invite family/partner to the consultations - A follow-up session after 12–18 months to revisit and evaluate the journey	- Offer from six months postdiagnosis to ensure enough diabetes experience to share in the group - Up to 12 participants in the groups - More time for peer-to-peer chatting - Slightly longer than two hours pr session and more spaced out - Regular follow-up sessions for social support - Giving participants access to answers to questionnaires to be able to compare between follow up time points
Content	- Site-specific information about the treatment they will receive with a more detailed checklist of the next steps in their own clinic - More space to discuss interacting factors that affect blood glucose values - Using shorter personalised questionnaires that can be used in the sessions to reflect or look back on previous months	- Possibility to add more personal nuances to the Diabetes Roadmap - Possibility to customise illustrations (tool 3) - More forward-looking activities, i.e., how to improve quality of consultations more concretely and seek out more support - Facilitators bringing up difficult topics such as sex, pregnancy and fertility or life expectancy - If group sessions replace clinical appointments, more space to pose questions around diabetes management, i.e., going through formal checklists of what they need to address in terms of self-management in the short, mid, and long term
Delivery	- If delivered virtually conversation tools should be sent to participants in advance - If continuity not possible with allocated nurse, the conversation tool provides continuity in a different way	- If group sessions replace clinical appointments, two HCPs would be preferable to ensure capacity to answer all questions posed during the session (in addition to the peer facilitator)
Outcomes	Participants from both interventions fed back that the questionnaires they completed were too lengthy. They suggested using shorter questionnaires that more accurately could assess their experience of a new T1D diagnosis and capture their experience of participating in the LADDER intervention.	

## Discussion

Adopting a mixed methods approach to assess the feasibility of LADDER allowed us to both assess the research processes quantitatively with regards to recruitment, retention, and data completion and at the same time explore the perception of attending the LADDER programme through interviews with participants. We were able to obtain meaningful quantitative data on the research processes. However, due to a lower than planned number of participants, a low response rate on the questionnaires, and unavailability of some clinical data, the quantitative data on clinical and psychosocial outcomes were insufficient. From the perspective of the participants the two LADDER interventions (one-to-one sessions and group sessions) were acceptable and feasible.

The recruitment rates differed substantially between the one-to-one consultations (79% and 71% in Denmark and the UK, respectively) and the group sessions (37%). While the recruitment target was reached in Denmark for the one-to-one consultations, it took longer than expected, and it was necessary to widen the inclusion criteria regarding diabetes duration. Similarly, this was needed in the UK. However, within the study period, it was not possible to recruit to target for either the one-to-one consultations or the group sessions. Consequently, randomisation had to be abandoned for the group sessions, resulting in an uncontrolled study design. Although most potential participants showed interest in taking part in the study, several either did not return to us after having received the study information or did not complete consent forms and hence were not recruited for the study. This occurred particularly often with those eligible for the group sessions. Recruitment differences may reflect varying levels of perceived need for support among the participant groups. Those eligible for the LADDER one-to-one sessions may experience heightened emotional and informational needs, as suggested by studies highlighting the psychological challenges and support needs associated with a T1D diagnosis (3, 39, 40). Additionally, the convenience of LADDER one-to-one consultations, which did not require additional clinic visits, may have further facilitated participation in the LADDER one-to-one consultations. In contrast, individuals with longer diabetes duration, who were eligible for the group sessions may have developed greater self-management confidence over time, potentially reducing perceived need for additional support (41). We hypothesise that recruitment difficulties were partly due to the emergence of the COVID-19 epidemic and working within the legacy of COVID-19, which affected the way in which clinics work. For example, the LADDER tools were developed to be used face-to-face and with more clinical visits being conducted virtually, this might hinder recruitment and engagement with the study. However, further research is needed to explore the underlying factors influencing recruitment disparities.

Retention rates for both interventions were high, with 78% and 80% of participants completing the LADDER one-to-one sessions, and 85% completing the group sessions. Among those who did not complete the interventions, most responded to the baseline questionnaire but did not attend any sessions, citing scheduling conflicts as a primary reason. Although group sessions were offered at various times—including afternoons, evenings, and weekends—limited availability may have constrained participation. These findings align with existing evidence that scheduling conflicts and competing demands are common barriers to attendance in group-based interventions (42). Future interventions could potentially benefit from greater flexibility in session scheduling to accommodate participants’ diverse needs.

The feasibility study showed that obtaining psychosocial outcome data was difficult. The completion of baseline data varied from 74% (one-to-one sessions in Denmark) to 95% (group sessions). The follow-up data were very limited, ranging from 62% at the three-month follow-up (group sessions) to 0% for the one-to-one sessions in the UK at the six-month follow-up. Although our PPI group helped us choose the questionnaires, the participants felt that the questionnaire was too long and not user friendly to complete and that the questions did not reflect what the intervention had contributed with. While low response rates have been reported in other studies (43), for our study, it may be that the use of established questionnaires not specifically tailored to capture their experiences, is not appropriae to evaluate a complex intervention in the adult-onset population. Therefore, developing a questionnaire that more accurately captures the unique experiences of the first part of life with adult-onset T1D might increase participants’ willingness to respond. Furthermore, we were not able to collect all the data on clinical outcomes as planned. For example, information about hypoglycaemia was not systematically reported and available from the electronic records and we did not ask participants to self-report these data. The quantitative data on clinical and psychosocial outcomes were, therefore, insufficient to estimate the standard deviation of any outcome because of the low response rate and lower uptake than expected. The change in study design to an uncontrolled study (the group sessions) and the small number of participants in the one-to-one study in the UK prevented us from making any comparisons. Therefore, the quantitative data can only be used to consider whether the measurements chosen are feasible for a future large study and as a description of the participants.

Our use of a mixed methods approach allowed us to explore participants’ perception of the LADDER programme. The qualitative findings suggest that the LADDER interventions may create a window of opportunity in which participants are supported to develop positive adaptive skills through processes such as reflection, validation, and sense-making triggered by the use of visual conversation tools within a supportive clinical context. Offering support to establish such skills is consistent with covering the gap in provision of psychosocial support highlighted by other studies (44, 45).

Previously, there has been a remarkably limited focus on providing psychological and social support in a systematic way to people with adult-onset T1D (7, 14, 46), which might be explained, in part, by the ongoing—but mistaken—assumption that a diagnosis of T1D occurs exclusively among children. Several studies have shown the importance of psychosocial support to children and their parents at the time of diagnosis (47, 48), but generally, systematic integration of psychosocial perspectives is poor in the routine care of the adult-onset population. In addition, another factor relates to the perception of lack of time in consultations, which means that psychosocial issues are often neglected, as it is deemed more important to focus on the biomedical aspects of diabetes management (49, 50). This common perception reflects a biomedical model of care (51, 52) and a prioritisation of the immediate procedures for survival. The findings from participant interviews suggest that when psychosocial aspects of living with T1D are addressed alongside biomedical management, participants experience greater coherence and relevance of care, which may, in turn, influence engagement with self-management education.

The divergence in experience between participants diagnosed four weeks ago and those with a slightly longer diabetes duration (up to 18 months) reflected the flux of the early period following diagnosis, as identified in our previous studies (3, 4). Our qualitative findings suggest that the two LADDER interventions may be experienced as relevant at different timepoints, insofar as participants engage with intervention components in ways that resonate with their evolving psychosocial needs following diagnosis. This suggests that, if aligned with service structures, the intervention could contribute to a clearer care pathway by supporting timely matching between individuals’ perceived needs and available types of care.

Conversely, participants suggested that having lived with T1D for at least six months was important for group sessions, as this appeared to enable comparison, mutual recognition, and meaningful exchange of experiences. In our study, participants were exposed to either LADDER one-to-one consultations or group sessions depending on their diabetes duration at the time of recruitment. Hence, we do not know if it would be desirable to be exposed to both interventions and, if so, how the timeline of these exposures would best be laid out.

Suggestion for optimisation such as including sensitive topics like sex and fertility should be possible to implement in a future study by equipping HCPs and the peer facilitator to actively mention such issues, which have also been requested in other studies (53, 54). We have added empty speech bubbles on the “Diabetes roadmap” to cater to individual differences and variety in experiences.

### Strengths and Limitations

A strength of the study is the mixed methods approach which assisted us in evaluating both the research processes and the potential perceived benefit of participating in LADDER including barriers and facilitators when delivering the interventions. Another strength is that we were able to recruit almost an equal number of male and female participants from five large diabetes clinics in two countries.

There are several limitations of the study that merits attention. While we were able to obtain sufficient qualitative data regarding the experiences of the benefits experienced by the participants who completed the interventions, we identified issues with recruitment, obtaining sufficient quantitative data, and consequently the controlled study designs.

Although five large diabetes clinics were involved in the study, it was not possible to reach the recruitment target within the allocated study time or with the intended inclusion criteria. This indicates difficulties in recruiting when the eligibility criterion is too narrow (i.e. < four weeks postdiagnosis), or having enough participants to conduct controlled trials in a small population, which is variable in numbers for each clinic. Potential participants might be sensitive to being asked to take part in randomised studies due to the chance of being excluded from a potential beneficial intervention. The low number of participants, the uncontrolled designs, the high attrition rate from questionnaire responses and the unavailability of sufficient clinical data negatively affected the internal validity of the study and prevented us from estimating an average treatment effect.

With these limitations consideration might be given to whether the assumption of controlled trials that an intervention is stable across contexts and that contextual factors can be controlled for analytically is well-suited for the evaluation of complex, context-sensitive interventions such as LADDER. One alternative consideration might be to employ Realist Evaluation (RE) to evaluate complex interventions such as LADDER in the adult-onset population. Using a RE approach could enhance the understanding of what it is about the LADDER interventions that does or does not work, for whom and under which circumstances (55) rather than merely exploring if it works. For example, the approach would take the flux of the first years with T1D, the interactions between HCPs and people with adult-onset T1D, and the differences in clinical settings into consideration when evaluating the outcomes of the interventions (56). A specific example of differences in clinical settings is, that four of the five diabetes clinics had been involved in the development of the LADDER interventions and might be positively predisposed to conduct the study. It would be relevant to explore if other diabetes clinics with less resources or lack of experience with psychosocial interventions might be less likely to engage with the LADDER programme or may face other challenges to adapt recruitment strategies or delivery to this specific setting.

Rather than producing effect sizes based on statistical comparisons RE uses a generative model to produce explanation about causal processes based on context-mechanism-outcome (CMO) configurations. CMOs are used to understand how certain intervention mechanisms are activated or not in various settings and what outcomes they produce (57, 58). The RE approach relies on data that can support theory-driven inference which are based on analytical and reasoned judgement. This process requires transparency and rigour through documentation of the analytical steps. While the qualitative data collected in this study provide insight into participants’ experiences and perceived value of LADDER, further realist analysis could better specify how particular intervention resources (e.g. visual conversation tools, peer exchange) interact with contextual features to trigger mechanisms such as reassurance, normalisation, or reflective sense-making, leading to psychosocial outcomes (59). For example, the participants reported that the LADDER interventions were beneficial, and on this basis, recommended their inclusion in care pathways. From a realist perspective, this suggests that under certain contextual conditions, LADDER may activate mechanisms that support psychosocial adaptation.

## Conclusion

Our findings from this mixed methods study feasibility testing two interventions in the LADDER programme in Denmark and the UK, respectively, showed that while the retention rates were high in both countries, recruitment was insufficient in the UK, leading to abandonment of randomisation. Response rates on psychosocial questionnaires were low overall, and some clinical data were unavailable. Together these challenges made it impossible to produce estimates of effect from the interventions. Qualitative data from interviews with participants suggested that the LADDER interventions, including the use of visual tools in consultations and group sessions, may support psychosocial adaptation by facilitating reflection, articulation of concerns, and shared understanding within supportive clinical and peer contexts.

Considering the LADDER programme is a complex intervention and the challenges of recruitment and obtaining quantitative data revealed by this study, employing a realist evaluation approach to a future larger study might be beneficial. Such an approach could help to understand what about the LADDER interventions that work or does not work, for whom, under which circumstances and why.

## Data Availability

The datasets presented in this article are not readily available because of potentially identifying and sensitive patient information. Data are available from the authors upon reasonable request. Requests to access the datasets should be directed to mdue0015@regionh.dk.
