# Supplementary figures and images for: Psychosocial support for adult-onset type 1 diabetes: the living with and adapting to type 1 diabetes programme - a cross-national feasibility study
Source: Front Clin Diabetes Healthc. 2026 Mar 17;7:1749766. doi: 10.3389/fcdhc.2026.1749766 (PMC13035499; doi:10.3389/fcdhc.2026.1749766)

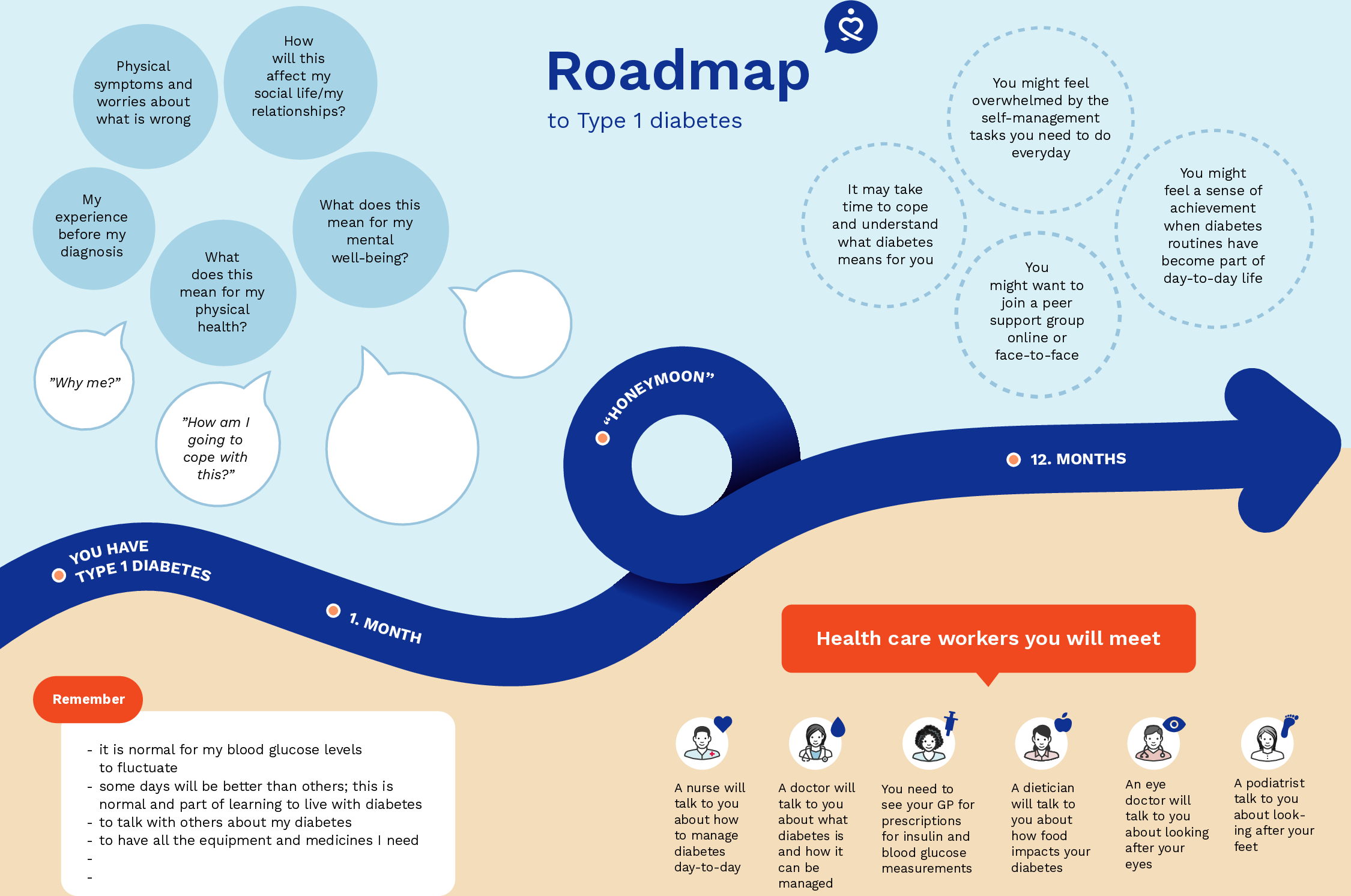

Supplement: Supplementary file 1 [file Image1.tif]

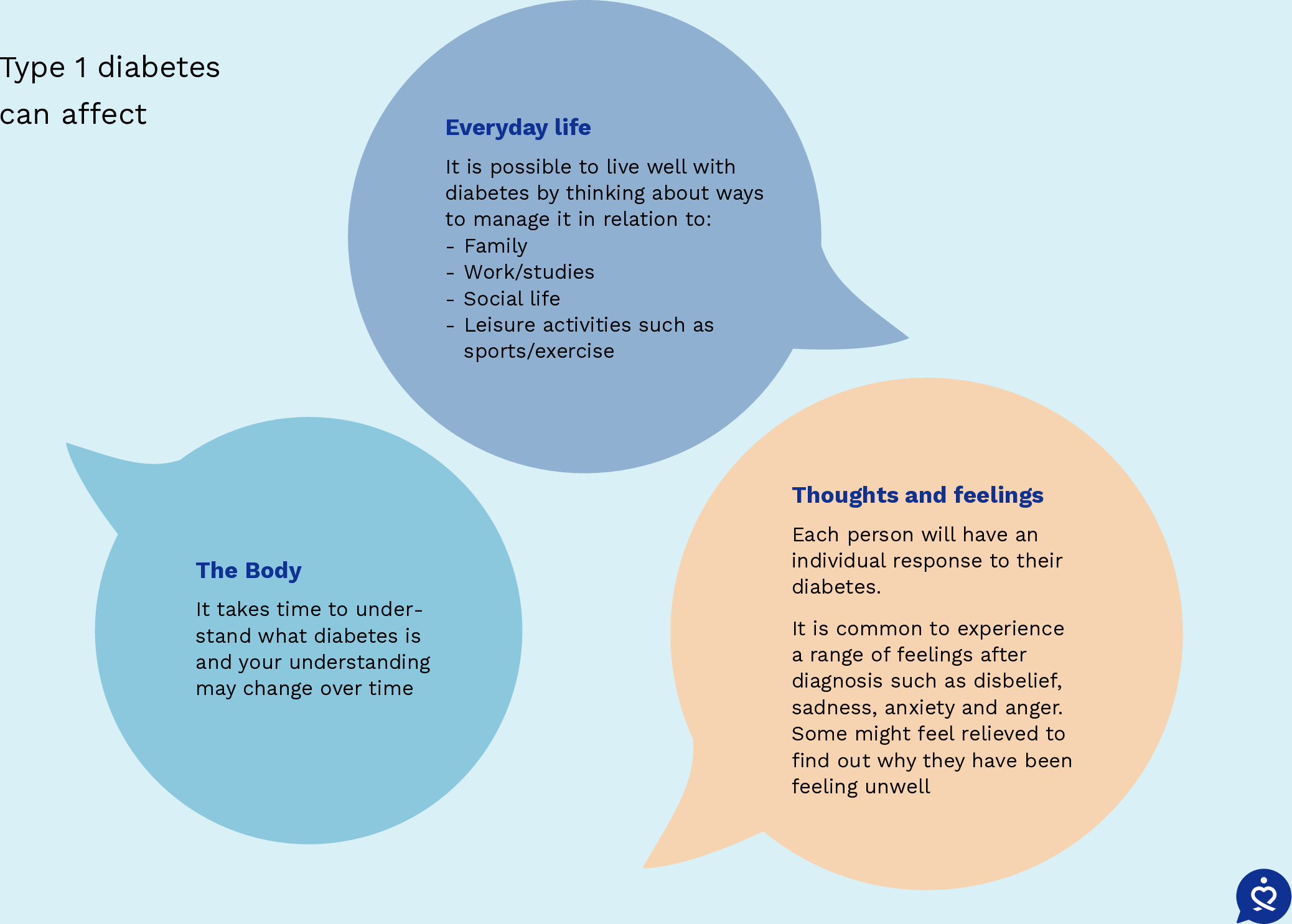

Supplement: Supplementary file 2 [file Image2.tif]

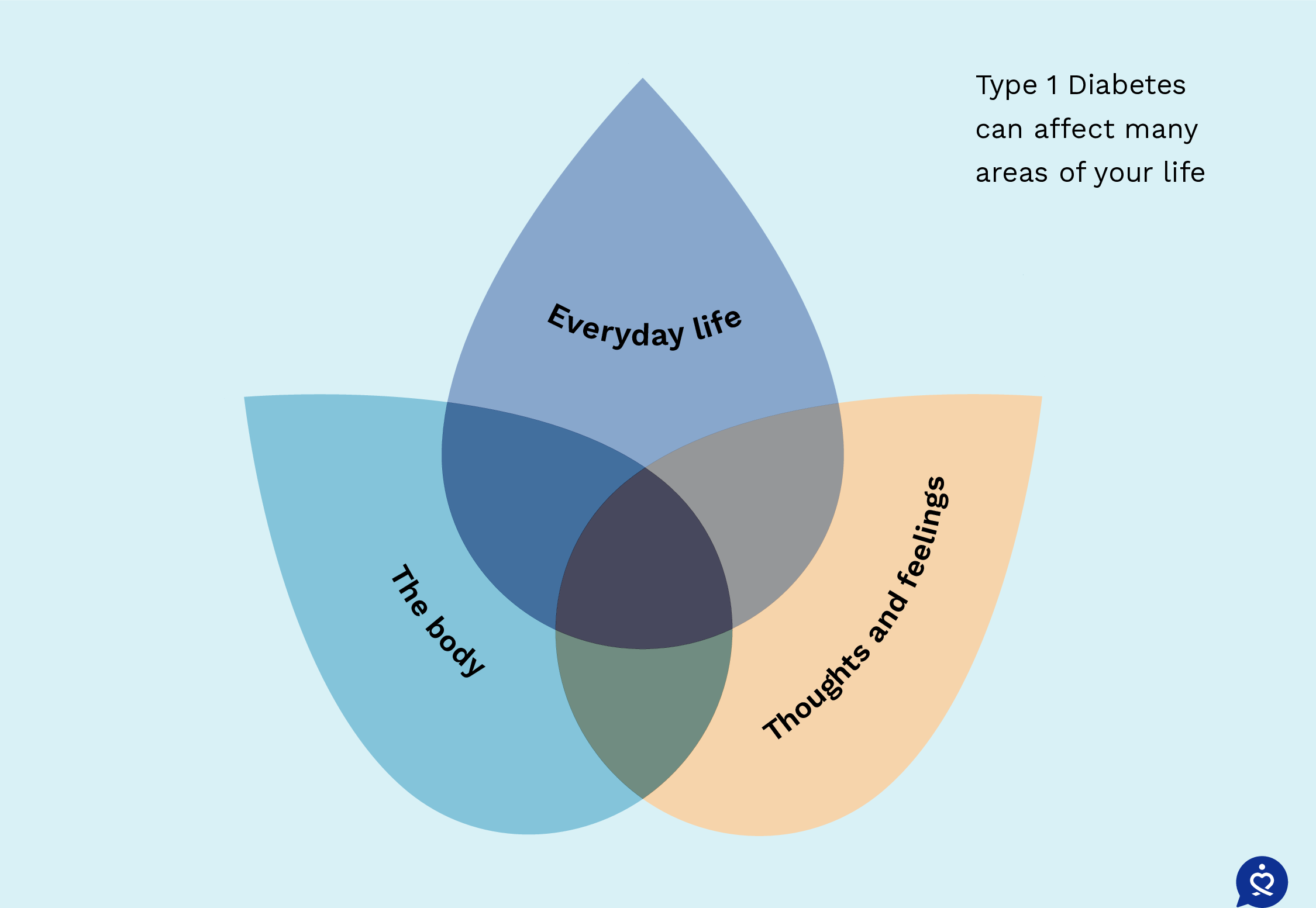

Supplement: Supplementary file 3 [file Image3.tif]

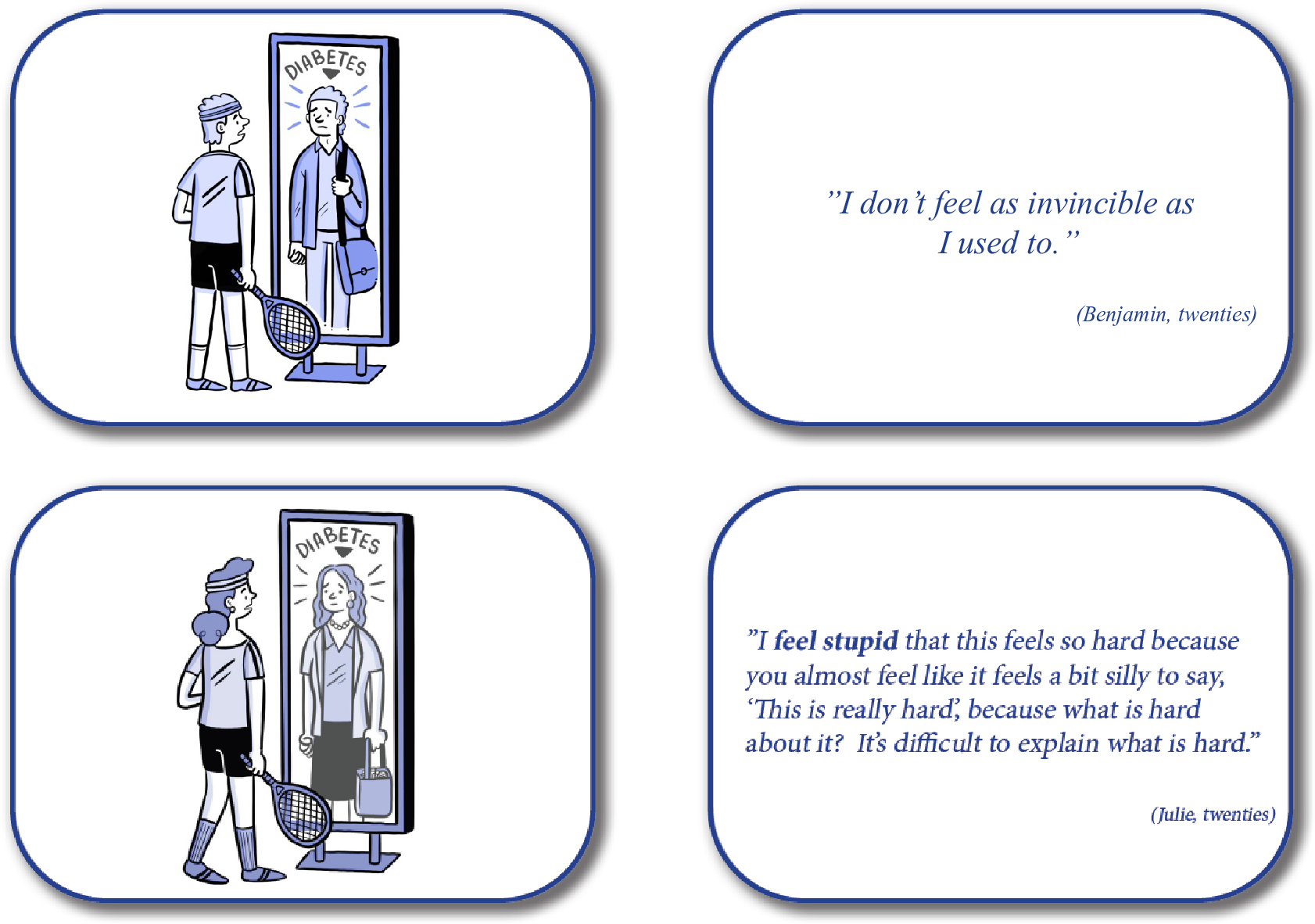

Supplement: Supplementary file 4 [file Image4.tif]

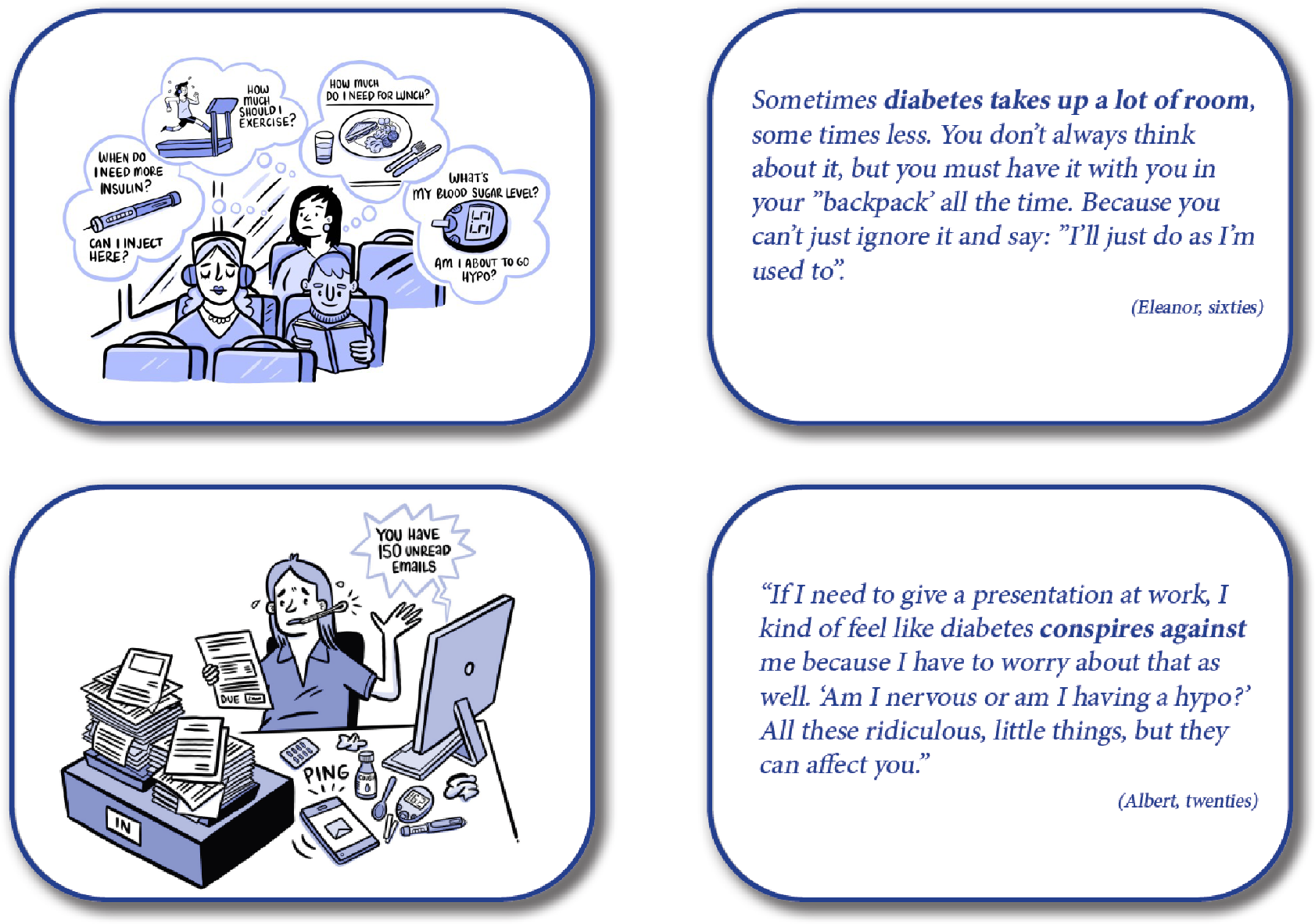

Supplement: Supplementary file 5 [file Image5.tif]
